# Development of an immunochromatographic strip test for the rapid detection of zearalenone in wheat from Jiangsu province, China

**DOI:** 10.1371/journal.pone.0175282

**Published:** 2017-05-10

**Authors:** Fang Ji, Mduduzi P. Mokoena, Hongyan Zhao, Ademola O. Olaniran, Jianrong Shi

**Affiliations:** 1 Key Lab of Food Quality and Safety of Jiangsu Province-State Key Laboratory Breeding/Key Laboratory of Control Technology and Standard for Agro-Product Safety and Quality/Key Laboratory of Agro-Product Safety Risk Evaluation (Nanjing), Ministry of Agriculture, China/ Institute of Food Quality and Safety, Jiangsu Academy of Agricultural Sciences, Nanjing, China; 2 University of KwaZulu-Natal, Discipline Microbiology, School of Life Sciences, Westville Campus, Private Bag X54001, Durban, South Africa; 3 Collaborative Innovation Center for Modern Grain Circulation and Safety, Nanjing, China; Seoul National University, REPUBLIC OF KOREA

## Abstract

A colloidal gold (ICS) test was developed for rapid detection of zearalenone (ZEN) in wheat samples. The mAb against ZEN was prepared in our laboratory and labelled with colloidal gold as a probe for the ICS test. The conditions were optimized and 30 nm colloidal gold nanoparticles were chosen for optimal performance. Millipore 135 was chosen as the NC membrane for its level of sensitivity. The optimum amount of coated antigen ZEN-OVA and anti-ZEN mAb was 0.5 mg/mL and 8 μg/mL, respectively. The ICS test, which has a detection limit of 15 ng/mL for ZEN, could be completed in 5 min. Analysis of ZEN in 202 wheat samples over three consecutive years revealed that data obtained from the ICS test were in a good agreement with LC-MS/MS data. This result demonstrated that the ICS test could be used as a qualitative tool to screen on-site for ZEN.

## Introduction

Mycotoxins are known as poisonous metabolites of fungi that exist on farmland or in the process of producing, transporting, handling or storing farm commodities and foodstuff [1,2]. In every year, up to 25% of grain crops are contaminated with mycotoxins, which results in substantial economic losses all around the world [3]. Zearalenone (ZEN) was found in 12.8% of the samples in the Jiangsu province, China [4]. Considering the substantial risks of public health and economic, it has received much attention among scientists who attempt to detect mycotoxins in foodstuff and fodder in recent years [5].

As a nonsteroidal mycotoxin which possesses estrogen-like activity, ZEN has been observed to be related to pubertas praecox, endometrial hyperplasia, endometrial neoplasms as well as carcinoma of uterine cervix [6]. Three carcinogens have been identified in ZEN by the International Agency for Research on Cancer [7,8]. Moreover, ZEN could be metabolized into α-Zearalenol (α-ZOL) with estrogenic activity that is three to four times higher than that of ZEN [9]. With the serious harm that ZEN poses to human and animal health, restrictions by law to the content permissible in grain and products, have been established by multiple international organizations. The domestic legal limit of ZEN is 60μg/kg according to the National Criterion [10]. Establishing simple, rapid and reliable methods for detecting mycotoxins is imperative for agriculture development and public health.

Several quantitative methods to test for the presence of ZEN are available. They include liquid chromatography-tandem mass spectrometry method (LC-MS/MS), high-performance liquid chromatography method (HPLC) as well as enzyme-linked immunosorbent assay method (ELISA). Nevertheless because of the complexity, strenuosity and long duration of HPLC and LC-MS/MS analyses, they are not applicable for routine high-throughput detection. Moreover, the usage of immunoassays is restricted by supporting facility in laboratories, and thus they are not appropriate for field service [11,12]. Consequently, there is an urgent need for proposing easy, efficient and inexpensive detecting methods to test mycotoxins in grain and corresponding products.

As a promising kind of one-step test that works considerably well, the immunostrip method utilizes nanoparticles exhibiting reddish color instead of routine enzymes as the detection marker, which is the typical characteristic of this method. Since it can be used to detect poisonous substances of small molecules in the field with the advantages of quick test results, low expense and reliability, the immune-chromatographic strip test (ICST) has drawn much attention from scientists who are engaged in food security research [13–15]. The test has been used to detect poisonous substances with low molecule weight such as AFB1 [16], DON [17,18], FB1 [19], OTA [20] and T-2 toxin [21]. Recently, ICST results to detect ZEN have been reported [11, 22].

Wheat is the staple crop in Jiangsu Province where the wheat florescence is accompanied with high humidity, pluvial period, warmth and weak daylight. This is in favor of the occurrence and progression of Fusarium head blight epidemics. The authors have reported the occurrence of ZEN in Jiangsu Province from 2010–2012 using LC-MS/MS method. It was demonstrated in this study that ZEN could be detected rapidly by ICS test in the wheat samples that were collected from several regions of Jiangsu Province from 2013 to 2015. As confirmed by LC-MS/MS test results, ICS test could be used by personnel with limited training to screen samples fast and selectively.

## Materials and methods

### Materials and chemicals

ZEN (100 μg/mL, dissolved in acetonitrile) was obtained from Sigma-Aldrich (China). Bovine serum albumin (BSA), sodium dodecyl benzenesulfonate (SDBS), goat anti-mouse IgG, mycose, polyethylene glycol (PEG, MW = 20,000), polyvinylpyrrolidone (PVP), sucrose, Tween-20 were purchased from the Sino-American Biotechnology Co. (Shanghai, China). The Anti-ZEN antibody was prepared in our laboratory and purified by Protein-G Sepharose Fast Flow Columns (Amersham, NJ, USA). In addition, other reagents were not inferior to analytically pure class and were purchased from DingGuo Biotech Co. (Nanjing, China). Absorbent pads, colloidal-gold particles (30 nm in diameter), conjugation pads, nitrocellulose (NC) membrane Millipore 135 and sample pads were purchased from Shanghai Kinbio Tech Co. (Shanghai, China).

Wheat samples were collected from several regions of Jiangsu province, southeast China, that are commonly heavily infected with *Fusarium* (S1 Table); The survey was conducted over 2013–2015 and a total of 202 wheat samples were collected from 13 counties during harvest. The type of these wheat samples are the main varieties of the locals, such as Yangmai, Ningmai, Huaimai and so on.

### Preparation of colloidal gold-MAb conjugates

Anti-ZEN MAb prepared in our laboratory was purified from mouse ascitic fluid using a caprylic acid and ammonium sulfate method [23], and purified further by affinity chromatography using Protein-G Sepharose Fast Flow columns (Amersham, NJ, USA). To produce a clear supernatant, the antibody was dialyzed against phosphate buffer (0.01 M, pH 7.4, PB) at 4°C for 48 h and centrifuged at 8,497 g for 10 min afterwards. 0.2 M K_2_CO_3_ or 0.1 M HCl was used to adjust the pH of colloidal-gold solution which was applied to binding antibody to 7.5. Anti-ZEN (100μg/ml, 0.8 mL) was mixed with colloidal-gold solution (10 mL) drop by drop while the solution was stirred softly to keep homogeneous. After 30 minutes of reaction time, PB containing BSA 10% (w/v) (1mL) which was used to cover redundant active sites on colloidal-gold particles was added into the solution for another 30 minutes of reaction. Subsequently, the solution was centrifuged at 4°C for 20min using the centrifuge force of 8,497 g. After removing the liquid supernatant with caution, 0.1 mL PBS (pH 7.4) of conjugate storage buffer which contained 0.05% NaN_3_, 1% BSA as well as 2.5% sucrose was used to re-suspend the pellets of colloidal-gold with antibody and finally the solution was stored at 4°C.

### Preparation of immunochromatographic strip

A typical immunostrip included three sample pads, pads for releasing and absorbing conjugates, cover tape, NC membranes as well as glass fiber.

The sample and conjugate pads were processed with 0.01M PBS (pH 7.4) which contained 1% BSA, 2.5% sucrose and 1% Tween-20 for 3h. Subsequently the pads were dried in vacuum at 37°C for 16 h.

Firstly, an absorbing pad was affixed onto the upper surface of the membrane sheet. Then a release pad was pasted onto the plate with a 5 mm crossover upon the NC member. Finally a sample pad was affixed onto the plate with a 7 mm crossover upon the release pad.

### The principle of immunochromatographic strip test

The theoretical basis of the ICS tests in this paper was competitive immunoassay. If sample solution contained no ZEN, anti-ZEN monoclonal antibody-gold conjugates could move into the Millipore 135 membrane with freedom and bind immobilized zearalenone-ovalbumin conjugates to form immune complex. If the concentration of ZEN was higher 15ng/mL in standard solution or 50 μg/kg in spiked samples, wheat sample solution could be imbibed from the sample pad due to the mechanism of capillary effect and all monoclonal antibody-gold conjugates on the release pad would be saturated. The saturated monoclonal antibody-gold conjugates which were imbibed up through capillary tubes could not be caught by ZEN-OVA in the test line. Subsequently the secondary antibody of goat anti-rabbit on the control line caught them and the result was manifested as an NC membrane with a single red line on it.

On the other hand, when the sample contained ZEN of less than 50 μg/kg, monoclonal antibody-gold nanoparticle conjugates that did not bind with ZEN were then captured by the ZEN-OVA complex in the test line and the negative result was manifested as a reddish test line.

### Detection of standard solution

With regard to standard liquid, 0.01 M PBS (pH 7.4) was used to dissolve ZEN into a series of concentration gradients (0, 5, 10, 15, 30, 60 ng/mL). The prepared ZEN solution was subsequently used to confirm the examination range of the strip.

#### Sample pretreatments and analysis

ICST and LC-MS/MS methods were used to determine the ZEN concentrations of extraction solutions of 202 samples in total. Firstly, a laboratory mill (IkaWerke, Staufen, Germany) was used to grind the wheat samples into 20 mesh fine powder.

Then 10 g fine powder of each sample was collected and mixed with 40 mL extracting solution (acetonitrile: water: acetic acid = 79:20:1v/v/v) and centrifuged for 30 min with the speed of 180 rpm [24]. After being centrifuged for 10 min with the speed of 3000 rpm, 0.5mL final extract of each sample was diluted with 0.5 mL extracting solution (acetonitrile: water: acetic acid = 79:20:1v/v/v). Subsequently, the mixture passed through a nylon filter which had the diameter and pore size of 13 mm and 0.22 μm separately and finally flowed into an auto sampler vial to be analyzed by LC-MS/MS [25–27].

With regard to the colloidal-gold immunochromatographic strip, 0.01 M PBS (pH 7.4) was utilized to dilute 0.5 mL filtrate of each sample into 2 times volume and 100 μL of above mentioned solution was added onto the sample pad. Five minutes later the result could be read. The tests on natural and spiked samples were both conducted in triplicate.

### Statistical analysis

All data were represented as percentage or mean ± relative standard deviation (RSD), respectively. Statistical analyzes were conducted with Microsoft Excel.

## Results and discussion

### Development and optimization of the strip test

As a rapid, quantitative detection immunosensor, the performance of the ICS test is affected by various parameters, such as the strip materials, sizes of colloidal-gold nanoparticles, concentrations of the antibodies labelled to colloidal-gold particles as well as pH of the colloidal-gold solutions for conjugation.

### Optimization of the strip materials

Vivid 170, Millipore 180, Millipore 135 and Sartorius CN 140 were tested in this study. Our results demonstrated that Vivid 170 and Sartorius CN 140 could not provide clear color, and Millipore 180 could not soak enough. Millipore 135 was selected to be used as the NC membrane, based on its best sensitivity in 5 min and low flow disturbance. Since Glass fiber filter paper SB06 could absorb more sample solution and heighten the color thus make the results clearer, it was selected to be used as the sample pad.

### Gold colloidal particles selection

Four kinds of colloidal-gold nanoparticles with the diameters of 21 nm, 25 nm, 30 nm and 40 nm, respectively were prepared for this study. Under a transmission electron microscope, the colloidal-gold solution was observed to be uniform and transparent.

Colloidal-gold nanoparticles with the diameter of 30 nm performed best in stability and the examination range of ZEN.

### Optimization of the concentration of coated antigens and antibodies for conjugated colloidal gold

The concentrations of coated antigens as well as monoclonal antibody-gold conjugates were optimized to enhance the sensitivity and accuracy of immune strips. The practical significance of these two methods lay on the feasibility that naked eyes could accurately distinguish the difference between positive results that exhibited a clear red line and negative results that performed differently. It was demonstrated that the optimum amount of coated antigen ZEN-OVA was 0.5 mg/mL, and the amount of anti-ZEN mAb was 8 μg for 1 ml of colloidal-gold.

### Detection limit of ZEN ICS test

**Fig 1:**
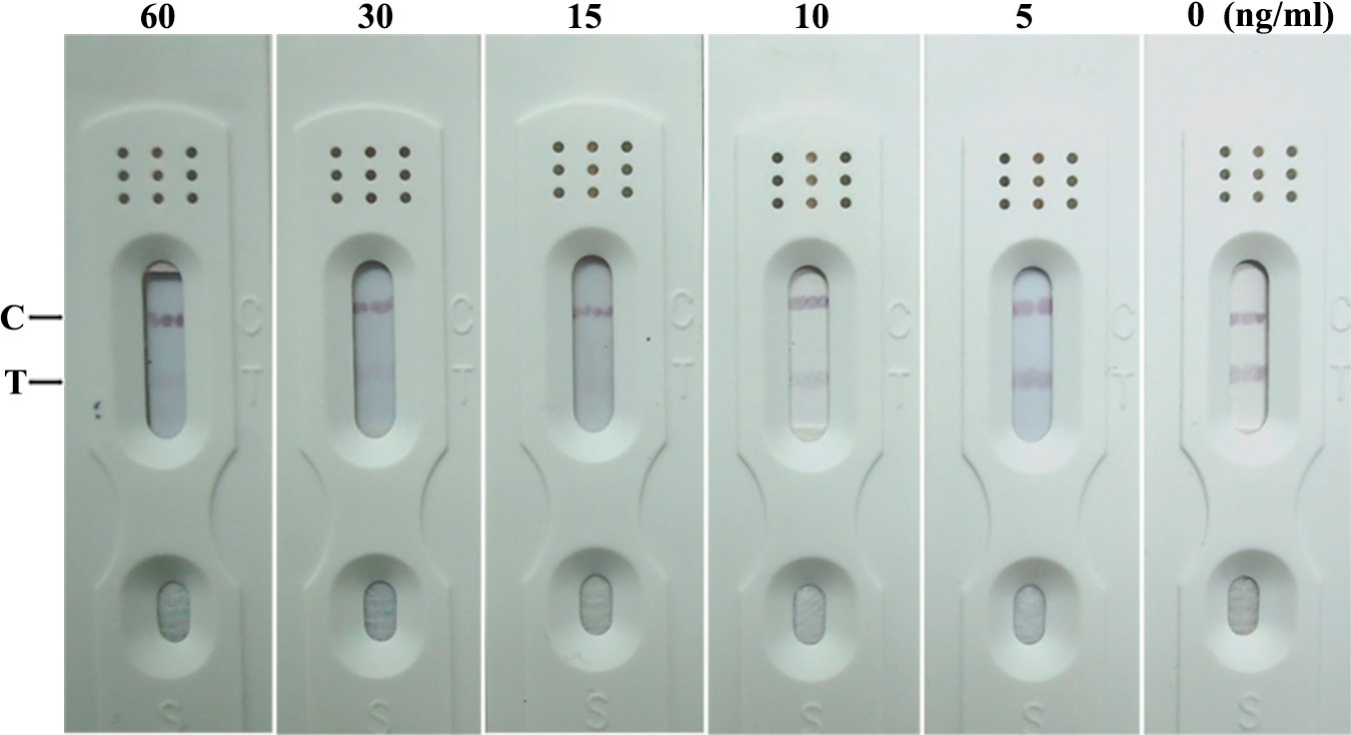
Cut-off limits for colloidal gold labeled test strip against ZEN (15 ng/mL).

10% acetonitrile / PBS was used to dilute ZEN stock into standard liquids of gradient concentrations which were added into sample pad afterwards as previously mentioned to determine the examination range of ZEN ICS test. The redness of test lines vanished after the concentration of ZEN reached 15 ng/mL which indicated that 15 ng/mL was the examination range of ZEN ICS test (Fig 1).

### Determining ZEN in spiked samples with colloidal-gold labeled antibody test strip

A drying step is required for many sample treatment procedures to concentrate the extract and to eliminate organic solvents that would adversely affect the antigen-antibody interaction. In our on-site detection method, the “drying step” was not suitable, as we did not have an instrument for drying. The extraction solution which included acetonitrile, water and acetic acid (79:20:1v/v/v) was used to guarantee that ZEN in the wheat samples was extracted completely. However, the established immunestrip system could not tolerate acetonitrile concentrations. Gradient dilutions were prepared with stock extraction solution and the 2-fold dilution was selected finally due to smaller effect on matrix and higher sensitivity to ZEN. In addition, it was demonstrated in previous studies that the extraction solution of acetonitrile and water co-extracted less interfering compounds in matrix compared with the solvent of methanol and water thus the former was used in this study [28, 29].

**Fig 2:**
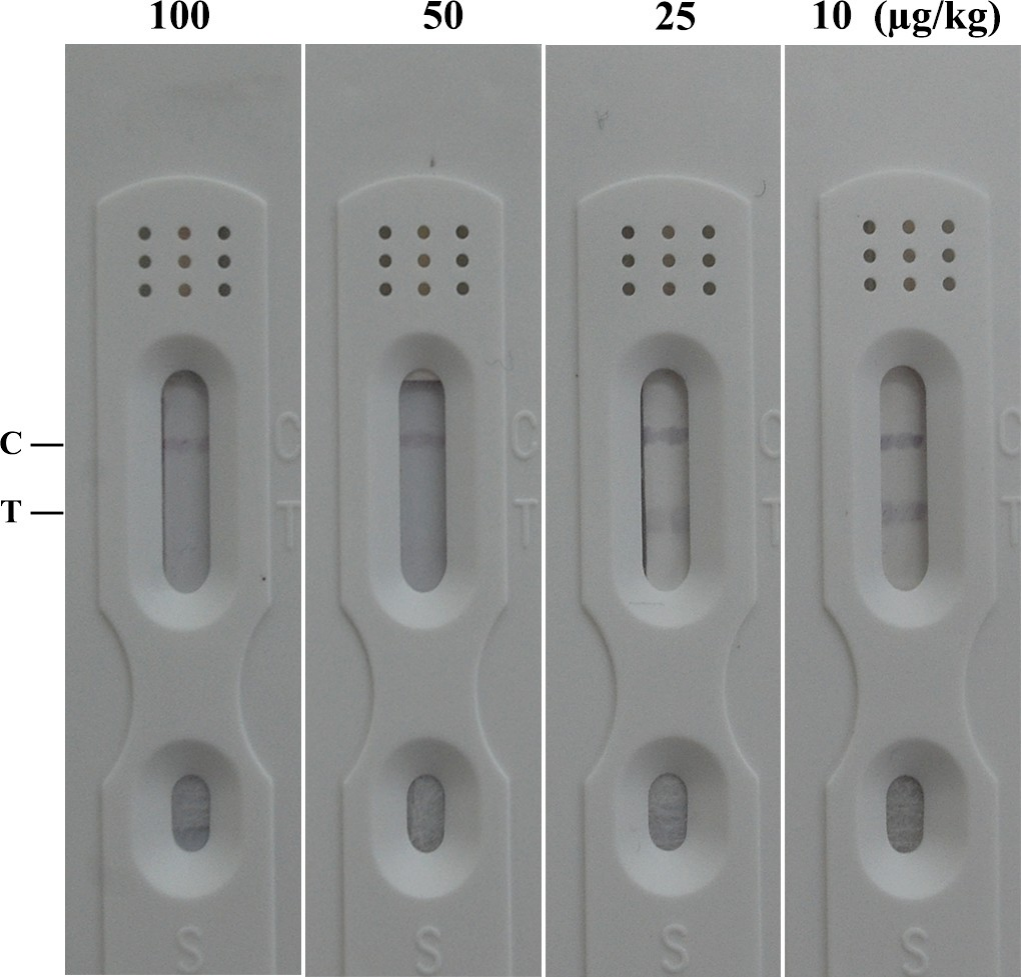
Sensitivity of spiked samples for ZEN.

As show in Fig 2, it was observed that 50 μg/kg of ZEN was sufficient to lead to positive results in spiked samples. The legal limit of ZEN in food is 60 μg/kg in China, thus the test strip was quite qualified for detecting ZEN domestically.

### Determining ZEN in spiked samples by strips and LC-MS/MS

The on-site ICS test was validated using 4 spiked samples, and confirmed by LC-MS/MS. All tests were conducted in triplicate. As Table 1 shows, there was a strong correlation between results from the ICS test and LC-MS/MS. The ICS test did not produce false-positive or false-negative results when the spiked concentration exceed 50 μg/kg.

**Table 1:** Results of ZEN analysis by LC-MS/MS and ICST in the spiked wheat samples.

Samples	Spiked (μg/kg)	LC-MS/MS (μg/kg)	ICST (n = 3)
1	10	9.12	─^b^, ─, ─^a^,
2	25	23.27	─, +, ─
3	50	46.21	+, +, +
4	100	96.52	+, +, +

a: Positive result, *T* line vanished.

b: Negative result, both *T* line and *C* line appeared clearly.

### Evaluation of cross-reactivity with other mycotoxins

**Fig 3:**
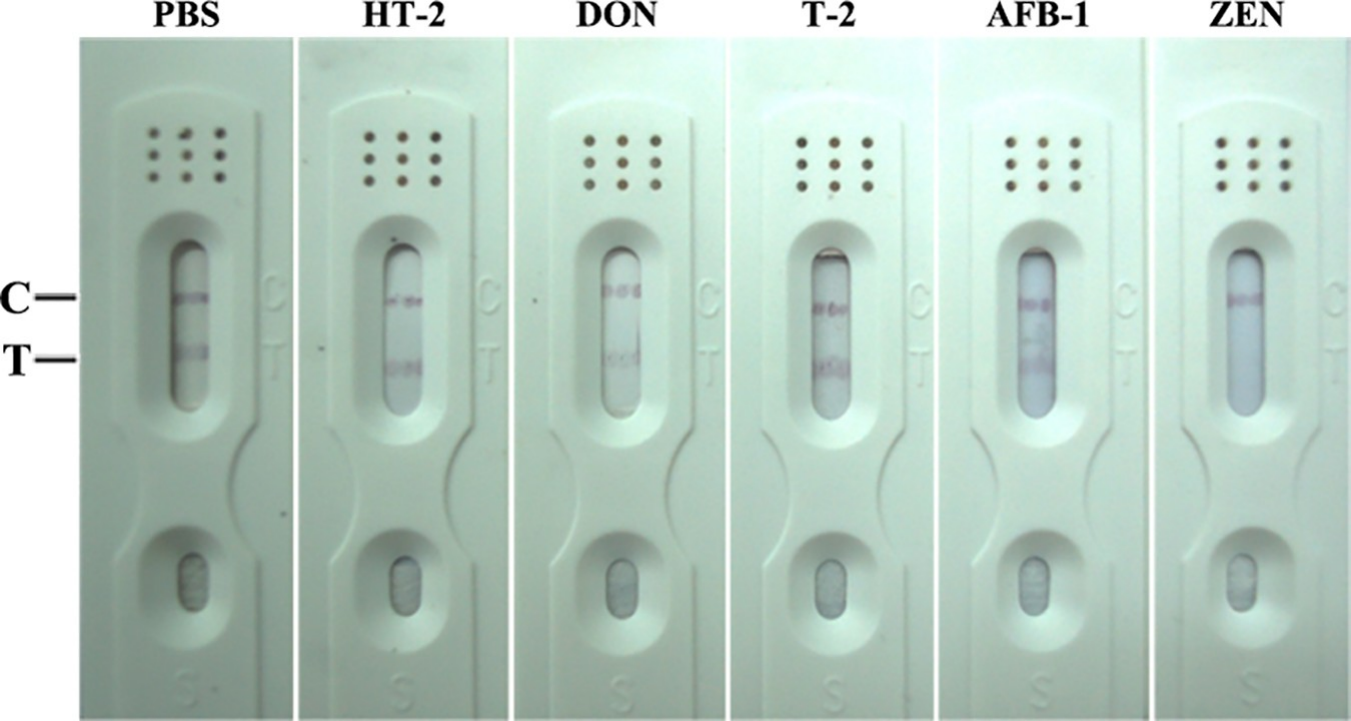
Cross-reaction with other mycotoxins. Concentration of these mycotoxins is 1μg/mL, respective.

**Fig 4:**
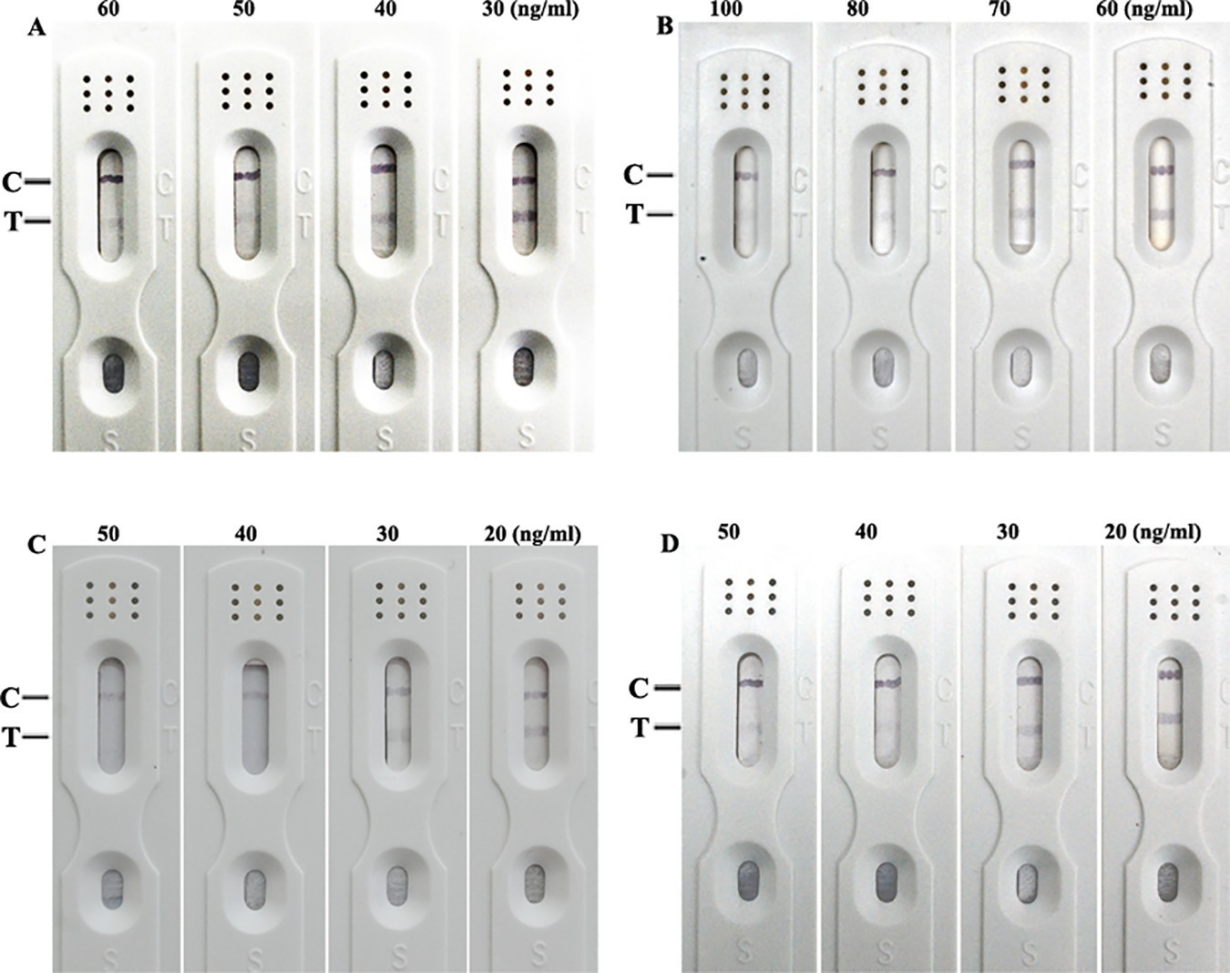
Cross-reaction with the metabolites of ZEN. (A: α-ZOL, B: β-ZOL, C: α-ZAL, D: β-ZAL)

To estimate the selectivity of the test strip, tests for other mycotoxins (HT-2 toxin, deoxynivalenol, T-2 toxin and aflatoxin B1) were performed. Samples were prepared in 10% acetonitrile/PBS at a fixed concentration of 1 μg/mL. Every sample was tested three times using the prepared ICTS. The color of the test line did not disappear, indicating that the cross-reactivity with these mycotoxins was negligible (Fig 3). In addition, the red test lines vanished while the concentrations of α-Zearalenol (α-ZOL), β-Zearalenol (β-ZOL), α- and β- Zearalanol (α- and β- ZAL; Fig 4) were not lower than 50 ng/mL, 80 ng/mL and 40 ng/mL, respectively. These results indicate that cross-reactivity with the metabolite of ZEN is not negligible.

This study aimed to propose a fast and non-instrumental immunochemical on-site test that could be performed outside the laboratory to examine ZEN concentrations of wheat and maize. In this study, the strip tests cross-reacted with α- ZOL, β- ZOL as well as α- ZAL and β- ZAL. ZEN is present in corn, wheat, oats and barley [30]. Following ingestion, it is rapidly absorbed in intestinal and transformed through liver metabolism into varied intermediates, including α- ZOL, β- ZOL, α- ZAL, β- ZAL as well as zearalanone [31, 32]. ZEN is not metabolized in wheat or maize, which would not affect the accuracy of the ICS test.

Huang *et al*. developed an ICST to detect ZEN and DON within 5 min, with a detection limit of ZEN of 60μg/kg [11]. They did not mention the cross-reaction with other mycotoxins. It was reported by Wang *et al*. that in spiked samples 60/500 ng/mL of ZEN/FB1 was sufficient to trigger positive reaction while in standard solutions 6/50 ng/mL of ZEN/FB1 was sufficient to trigger positive reaction. In addition, they did not find any cross-reactions between antibodies and FB1, DON or AFB1, but they did not mention the metabolism of ZEN. Their reaction could be completed in less than 15 min, while our strip could detect ZEN in 5 min [22].

### Stability of ICS

The assembly that completed ICS was stored at room temperature and 4℃ for 12 months. Desiccant of silica gel was used in plastic bags which contained the assembly to avoid moisture. During storage, the ICS test was applied to the ZEN solutions (0, 10, 15, 30 ng/mL) every day for 1 week, and then for an interval of 7 days, and a gap of 30 days for the remaining 11 months. It is observed in Table 2 that the test line of the ICS stored at room temperature could become red with ZEN ≥ 15 ng/mL for a month, and at 4℃ the strip could accurately identify the positive samples over a period of 12 months. Therefore, it was concluded that the periods of validity and corresponding preservation conditions of ICS developed in this study were 12 months in 4℃ or 1 month at room temperature respectively.

**Table 2:** Results of stability of the ICS test.

Days	store in room				store in 4℃			
	concentration of ZEN (ng/mL)				concentration of ZEN (ng/mL)			
	0	10	15	30	0	10	15	30
1d	─^a^	─	+^b^	+	─	─	+	+
2d	─	─	+	+	─	─	+	+
3d	─	─	+	+	─	─	+	+
4d	─	─	+	+	─	─	+	+
5d	─	─	+	+	─	─	+	+
6d	─	─	+	+	─	─	+	+
7d	─	─	+	+	─	─	+	+
14d	─	─	+	+	─	─	+	+
30d	─	─	+	+	─	─	+	+
60d	─	─	─	+	─	─	+	+
90d	─	─	─	+	─	─	+	+
120d	─	─	─	+	─	─	+	+
150d	─	+	ND	─	─	─	+	+
180d	─	ND	─	─	─	─	+	+
210d	─	─	─	─	─	─	+	+
240d	ND	ND	ND	─	─	─	+	+
270d	ND	ND	ND	ND	─	─	+	+
300d	ND	ND	ND	ND	─	─	+	+
330d	ND	ND	ND	ND	─	─	+	+
360d	ND	ND	ND	ND	─	─	+	+
390d	ND	ND	ND	ND	ND	ND	ND	ND

a: Positive result, *T* line vanished.

b: Negative result, both *T* line and *C* line appeared clearly.

ND: Not detected (the limit detection of ZEN is 15 ng/mL).

### Occurrence of zearalenone in wheat grown in different seasons and regions of Jiangsu province

202 wheat grain samples were analysed and the results are summarized in Table 3. In 2013, average, 40% of the samples is positive, while the samples with ZEN concentration higher than 50 μg/kg is 5.5%; In 2014, 49% of the samples is positive and 13.7% of which exceed 50 μg/kg; In 2015, the positive samples and the samples with ZEN concentration over 50 μg/kg are higher than in the past two years. As Table 3 shows, when the concentration of ZEN is more than 50μg/kg, the analysis results from ICS were highly consistent with those from LC-MS/MS.

**Table 3:** Occurrence of zearalenone in different seasons and regions of Jiangsu province analysis by LC-MS/MS and ICST.

			HPLC-MS/MS					ICST
Growing seasons	Region	n	positive	range (μg/kg)	average	medial	number of sample that concentration higher than 50μg/kg	positive
2013	Southern Jiangsu	25	11	6.49–49.62	25.7	25.66	0	0
	Central Jiangsu	16	10	15.89–110.03	36.98	35.95	2	2
	Northern Jiangsu	25	4	24.29–52.85	31.56	24.54	1	1
2014	Southern Jiangsu	26	12	16.03–55.22	36.8	40.3	2	2
	Central Jiangsu	15	9	15.15–194.32	50.21	31.76	2	2
	Northern Jiangsu	25	10	15.02–153.78	55.56	50.31	5	5
2015	Southern Jiangsu	24	12	25.1–142.0	50.6	36.9	3	3
	Central Jiangsu	21	11	27.7–114.1	48.9	38.7	2	2
	Northern Jiangsu	25	15	26.7–307.3	106.9	73.1	10	10

On the other hand, the occurrence of ZEN in Central Jiangsu is higher than Southern and Northern Jiangsu in 2013 and 2014, and this is consistent with our previous report [4,33]. In 2015, ZEN was present highest in Northern Jiangsu where there is higher rainfall between flowering and harvesting in that year. Kharbikar *et al*. reported that ZEN can be increased greatly by rain after florescence. Moreover, what is important is that even in wheat with serious FHB infection, ZEN level keeps quite low without a humid late season [34].

## Conclusion

This paper demonstrates a colloidal gold test that providing important high-throughput way for ZEN monitoring. The proposed ICS test method showed many advantages such as low cost, easy fabrication, short assay time, and user-friendliness. For the determination of ZEN, the mAb exhibited high affinity for ZEN and its metabolites and no cross-reactivity with aflatoxins B1, T-2, DON and HT-2. The effects of the influencing factors such as the strip materials, sizes of colloidal-gold nanoparticles, concentrations of the antibodies labelled to colloidal-gold particles and the pH of the colloidal-gold solutions for conjugation were verified. Under optimal conditions, the detection limit of the test was 15 ng/mL in standard samples and 50 ng/mL in spiked wheat samples, respectively. The whole detection process was accomplished within 5 min without complicated sample pretreatments. The results for detection of 202 wheat samples were in good accordance with the HPLC-MS/MS method and and demonstrated its application potential for ZEN monitoring on-site.

## Supporting information

S1 Table The source and type of wheat samples. (XLSX)
